# Challenges faced and coping strategies used by Malaysian International Medical Graduates surviving internships: A Qualitative Study

**DOI:** 10.1371/journal.pone.0323556

**Published:** 2025-05-09

**Authors:** Syuhada Hamzah, Aizuddin A. N., Sharifa Ezat Wan Puteh, Siti Rahayu Mat Husin, Mohamed Shazwan Zailani, Abdul Aziz Harith

**Affiliations:** 1 Department of Public Health Medicine, Universiti Kebangsaan Malaysia, Kuala Lumpur, Malaysia; 2 Medical Development Division, Ministry of Health, Putrajaya, Malaysia; 3 Occupational Health Research Centre, Institute for Public Health, Shah Alam, Malaysia; Ataturk University, Faculty of Pharmacy, TÜRKIYE

## Abstract

**Background:**

Malaysian International Medical Graduates (MIMGs) faced unique challenges during their medical internships in Malaysia, impacting their training experience and overall well-being. This study aims to explore these challenges and identify the coping strategies MIMGs employ to navigate their internships successfully. By understanding these factors, the study seeks to provide insights for improving the internship experience and reducing attrition rates among MIMGs.

**Methods:**

A qualitative research design was employed, utilising in-depth interviews with eleven MIMGs to gather detailed insights into their internship experiences. Thematic analysis was used to identify and analyse common themes and subthemes related to individual, training system, and working environment challenges and the strategies employed to cope with these challenges.

**Findings:**

The study identified three main themes, eight subthemes of challenges faced by MIMGs, and thirteen subthemes for coping strategies to survive an internship. The themes are individual challenges, training system issues, and working environment difficulties. Individual challenges included high levels of stress and burnout due to long working hours, heavy patient loads, and emotional tolls. Training system issues encompassed bureaucratic hurdles, inadequate infrastructure, and low financial incentives. The challenges of the working environment involved difficulties with cultural adaptation, hierarchical structures, and language barriers. MIMGs employed various coping strategies, with peer support emerging as a powerful tool, along with resilience, self-care practices, and proactive communication.

**Conclusion:**

This study provides valuable insights into the challenges and coping strategies of MIMGs during their internships in Malaysia. The results suggest that targeted interventions and systemic changes are necessary to support MIMGs effectively and enhance their training experience. Future research should explore these issues further, with larger samples and in different contexts, to develop comprehensive strategies for improving medical internships globally.

## Introduction

Medical internships in Malaysia serve as a critical phase in training newly graduated doctors, bridging the gap between academic medical education and full professional practice [1,2]. This two-year internship is mandatory for all new medical graduates and is designed to provide hands-on experience in various medical disciplines, including internal medicine, general surgery, paediatrics, obstetrics and gynaecology, orthopaedics, and one of four elective disciplines: emergency medicine, anaesthesia and perfusion, psychiatry, or family medicine [1,2]. The internship, or “housemanship,” as it is commonly referred to in Malaysia, takes place in government hospitals, teaching hospitals, and military hospitals, which are the primary training infrastructure due to their high patient volume and diverse case mix [3,4]. The structured program ensures that interns rotate for at least four months in each different department, gaining comprehensive exposure to various medical specialities [1,2]. This period is not only a time for skill development but also for acclimatisation to the rigours and responsibilities of medical practice, including patient care, clinical decision-making, and the administrative aspects of healthcare delivery [1].

The Malaysian healthcare system, characterised by its dual public-private structure, places significant emphasis on the public sector for medical training [5,6]. Government hospitals are funded by the Ministry of Health, teaching hospitals by the Ministry of Higher Education, and military hospitals by the Ministry of Defense, which provide the necessary infrastructure and resources for internship training [6,7]. These hospitals are often bustling with activity, presenting a high-pressure environment where medical interns must quickly adapt to the pace and demands of clinical work [7–9]. The training program is supervised by specialists or senior medical professionals who mentor and evaluate the interns, ensuring they meet the required competencies before progressing to fully licensed medical practitioners [7,10]. This period of training is crucial, as it serves to standardise medical practices and ensure that all doctors, regardless of their educational background, are competent to deliver safe and effective patient care [1].

In recent years, the Malaysian healthcare system has seen an influx of Malaysian International Medical Graduates (MIMGs), Malaysian citizens who have obtained their medical degrees from abroad universities [5,11–13]. The trend has shown an increase since the medical program moratorium was introduced in 2010 [13,14]. These graduates face unique challenges as they transition into the local internship program [15,16]. Unlike their locally trained counterparts, MIMGs often must navigate significant differences in medical protocols, cultural expectations, and administrative procedures [16,17]. The integration of MIMGs into the Malaysian healthcare system is acceptable, given the increasing number of Malaysians pursuing medical education abroad due to limited local medical school placements [13,18]. Therefore, the internship period becomes a critical juncture for MIMGs to adapt to the local healthcare environment, refine their clinical skills, and understand the nuances of the Malaysian healthcare system [14,17,19].

The process of medical internship in Malaysia is not without its challenges [20,21]. High patient loads, long working hours, and the need for quick adaptation to clinical environments can be overwhelming for interns [22]. Additionally, the hierarchical structure within Malaysian hospitals means that interns are often at the bottom of the chain, requiring them to navigate the dynamics of senior-junior relationships effectively [10,23,24]. Despite these challenges, the internship period is viewed as a rite of passage, essential for building the resilience, competence, and confidence needed for a successful medical career [17,25,26]. Ensuring that all interns, particularly MIMGs, receive adequate support and mentorship is vital for maintaining the high standards of healthcare delivery in Malaysia [7]. As Malaysia continues to develop its medical infrastructure and training programs, understanding and addressing the unique challenges faced by MIMGs will be crucial in ensuring a robust and effective healthcare workforce for the future [7].

Review from other countries: International Medical Graduates (IMGs) who have studied abroad and returned to their home countries face significant challenges as they undertake medical internships [27,28]. These challenges come from differences in medical education systems, cultural practices, clinical protocols, and workplace environments [27,29,30]. Despite these hurdles, IMGs often employ several common coping strategies that enable them to navigate and succeed in their demanding internship programs [27]. Key among these strategies are developing resilience and adaptability, actively seeking feedback, maintaining a healthy work-life balance, and engaging in continuous professional development [31–33].

IMGs, especially in Australia, Singapore, the United States, and the United Kingdom, employ common coping strategies to navigate and succeed in their medical internships [31–33]. Developing resilience and adaptability, actively seeking feedback, maintaining a healthy work-life balance, and engaging in continuous professional development are key to their success [32]. By cultivating these qualities and leveraging available resources, IMGs can overcome the challenges of adapting to new healthcare environments and build fulfilling careers in their respective countries [32,34]. These strategies enhance their professional performance and contribute to their overall well-being, enabling them to thrive in the demanding field of medical practice [32].

This study explored the challenges and coping strategies of MIMGs during their internship training in Malaysia. The goal is to prompt action from medical interns’ stakeholders in Malaysia, aiming to reduce dropouts or training extensions among MIMGs. By identifying their coping strategies, the study aims to offer insights into their challenges and suggest ways to improve support mechanisms in the program. This will enhance the overall effectiveness and efficiency of Malaysia’s medical internship training system, ensuring better preparation and support for MIMGs as they progress towards becoming full medical practitioners.

## Methods

We conducted a phenomenology qualitative study design with semi-structured interviews to explore MIMGs’ experiences with challenges faced and coping strategies for surviving an internship in Malaysia. In this study, we selected participants using purposive sampling. SRMH and MSZ identified 100 MIMGs those fulfill the inclusion criterias to be interview; Malaysian citizen, international medical graduates, employment started in 2018, contract medical doctor, and completed a medical internship not more than 3 months during the selection date, 1 April 2022. SH sent the invitation email to them. The Participant Information Sheet (PIS) and google form link contains basic demographic survey, and consent statement link were attached together.

Twenty [19] interested participants completed the online consent form were contacted by SH to schedule an interview date. Nevertheless, because of data saturation, interviews were conducted with only eleven [11] participants. In addition, we also obtained verbal consent prior to the interview session. Interview sessions held between 11 April until 30 July 2022. Before conducting the interviews, the AA and SH informed the participants about the study’s purposes and procedures, stating that the interviews would be audio-recorded.

They were informed that the interview content would be anonymised for research purposes only and that they could terminate their interview at any time. They were also informed that all data would be saved on a password-protected computer designated by the SH and would not be used for any purpose other than for this research. The researchers further informed the participants that they would discard the data after reporting the study findings. There were no conflicts of interest among the researchers in this study.

During the interview, SH and AA conducted semi-structured interviews using hybrid modes; physical and virtual. The convenient meeting mode was chosen by the participants. Each interview lasted between 30 and 60 minutes, and audio recordings were captured using Zoom applications. The semi-structured interview included inquiries about the challenges faced both before and during the internship, as well as strategies for enduring the internship and overcoming challenges using these lead and prompt questions tabulate in Table 1:

**Table 1:** Semi-structured Interview Question.

Lead Questions	Prompts
1. As international medical graduates, what challenges did you face before and during the internship?	1. Did you think of any personal challenges that you can’t avoid? 2. Did you think it is related to your medical training at university? 3. Do you think it is related to the internship system or working environment?
4. How did you cope to survive the internship?	1. What motivated you to complete the training? 2. How does your organization support you?

Simultaneously, we transcribed the captured audio recordings and developed the themes. We continued the interviews when the data interviews failed to reveal any new information or patterns that could potentially provide further insights.

Three criteria were adopted for this study to ensure research rigour. First, credibility was checked through member checking. Each participant received a copy of the interview transcript for review and verification to reflect their sharing during the interview. All researchers responded via email and validated the sharing. Second, an audit trail record was kept, listing the process from obtaining ethical approval to completing the study. It confirmed the study’s findings based on collected data rather than personal beliefs or interests. Furthermore, transferability was addressed by providing a rich, thick description of the research context, participants, and procedures. This level of detail enables other researchers to determine the applicability of the findings to similar contexts or populations, thereby enhancing the generalizability of the study’s conclusions.

We analysed the data using a six-phase thematic analysis to determine the meaning of participants’ experiences from interview transcripts [35,36]. The six phases include data familiarisation, identifying codes, finding themes, reviewing themes, defining and naming themes, and report writing [35]. In step 1, the data from the transcription were identified and familiarized. We repeated the “data familiarizing” process to increase our familiarity with each data point prior to the coding process. In step 2, we identified initial codes for challenges encountered before and during the internship, namely “emotional effects,” “learning challenges,” “training challenges,” and “work challenges.” Subsequently, initial codes for coping strategies to survive the internship, such as “intrinsic motivation,” “psychosocial support,” and “extrinsic motivation.” In step 3, the ongoing process of examining the codes and organising them into broader themes continued. We further reviewed the identified initial codes, which resulted in the emergence of preliminary themes named “individual challenges,” “training system challenges,” working environment challenges,” “individual coping strategies,” “coping with the training,” and “coping with the working environment.” Step 4 involved reviewing and refining the preliminary themes identified through data coding, which represented the context of challenges faced and coping strategies within the entire data set. In step 5, the final themes and subthemes were defined and named. Finally, in step 6, results based on the established themes were written.

This study is part of main study by SH, AA and SEWP about Factors Influencing Medical Internship Performance in Malaysia. We received approval from the Research Ethics Committee UKM (FF-2021–290) and the National Medical Research Ethics Committee of the Ministry of Health Malaysia (NMRR-21-1404-58617-IRR).

## Results

The profiles of the participants interviewed in this study are tabulated in Table 2. The participants’ ages range from 24 to 27 years old, with a majority being 25 or 26. The gender distribution includes seven females and four males, representing various ethnic groups in Malaysia; six are Malay, two are Indian, and one is Chinese. These participants have completed their medical education abroad; four are studying in Indonesia, two are in Jordan, two are in Ukraine, and one is each in Russia, India, and Egypt. This diversity highlights the participants’ varied backgrounds regarding demography and international educational experiences.

**Table 2:** The Profiles of Study Participants.

Participant ID	Age	Gender	Race	Country of University
MIMG1	26	Female	Malay	Indonesia
MIMG2	25	Female	Chinese	India
MIMG3	26	Female	Malay	Jordan
MIMG4	26	Male	Malay	Indonesia
MIMG5	25	Female	Malay	Russia
MIMG6	25	Male	Malay	Indonesia
MIMG7	25	Female	Malay	Egypt
MIMG8	27	Male	Malay	Jordan
MIMG9	24	Male	Indian	Ukraine
MIMG10	25	Female	Indian	Ukraine
MIMG11	25	Female	Malay	Indonesia

These in-depth interviews revealed three themes for each context: challenges faced by MIMGs before and during the internship, and coping strategies used by MIMGs to survive or complete the internship in Malaysia. The themes and subthemes are summarised in Table 3.

**Table 3:** Thematic Analysis on MIMGs Challenges and Coping Strategies Surviving Internship.

Theme	Subtheme
**Context 1: Challenges faced prior to and during the internship**	
1. Individual challenges	1. Stress and burnout 2. Discrimination
2. Training system challenges	3. Policy hurdles 4. Poor infrastructure 5. Low incentives 6. Non-compliance with flexi-hour
3. Working environment challenges	7. Communication barrier 8. Different work culture
**Context 2: Coping strategies to survive internship**	
1. Individual coping strategies	1. Self-motivated 2. Flexibility 3. Seeking support system 4. Continuous learning 5. Personal self-care
2. Coping with the training system	6. Effort to understand the policy 7. Work with less 8. Part-time work 9. Time management
3. Coping with working environment	10. Understand organizational culture 11. Adhere to local clinical practice guideline 12. Sensitivee about high public demand 13. Improve communication skill

## Challenges faced prior to and during the internship

**Fig 1:**
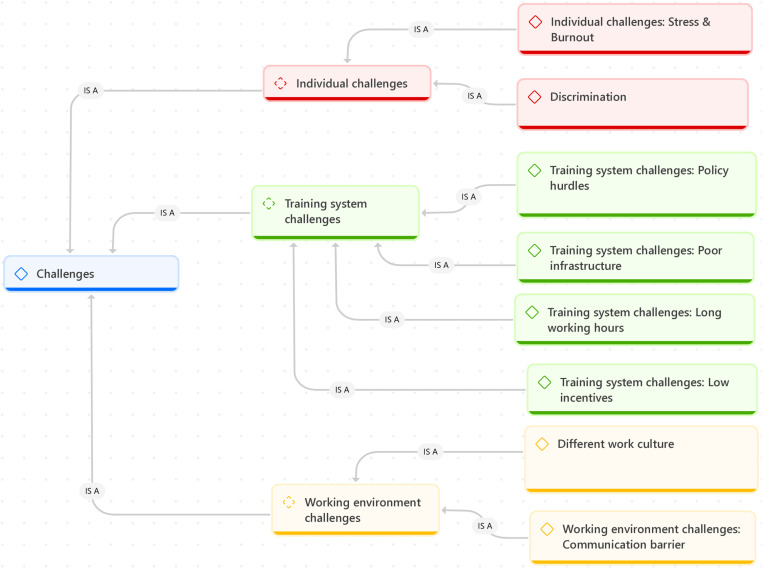
Thematic diagram illustrating challenges faced by Malaysian International Medical Graduates (MIMGs) during medical internships.

MIMGs face numerous challenges during their internships, especially when adapting to new healthcare systems. These challenges can be categorised into three themes named individual challenges, training systems, and working environment challenges (Fig 1).

### Theme 1: Individual challenges

Two subthemes about individual challenges among MIMGs were “stress and burnout” and ‘discrimination.’

#### Subtheme 1: Stress and Burnout.

The demanding nature of medical internships leads to high-stress levels and burnout among MIMGs. These pressures arise from various factors, such as heavy patient loads and the emotional challenges of patient care. The impact of these stressors can be significant, impacting not only the interns’ physical and mental well-being but also their professional performance and the quality of care they deliver.

The heavy patient loads that MIMGs must manage contribute significantly to their stress levels. Interns are often responsible for numerous patients, each requiring detailed attention and care. The sheer volume of patients can be overwhelming, making it difficult for interns to provide the level of care they aspire to. MIMG 6 discussed the emotional impact of managing critical cases and patient deaths:

“Losing a patient or dealing with critical cases back-to-back was emotionally draining. It affected my ability to stay focused and positive.” (MIMG 6)

Constant exposure to severe and often distressing medical conditions can be emotionally challenging for interns. Dealing with patient deaths, critical illnesses, and the associated family demands adds a layer of stress that is difficult to manage, especially for those in the early stages of their internship.

The absence of adequate support from senior staff and mentors exacerbates the stress and burnout experienced by MIMGs. When interns do not receive sufficient guidance and assistance, they can become overwhelmed by their responsibilities. This lack of support can lead to helplessness and frustration, further intensifying their stress. MIMG 11 highlighted the challenges of working without adequate support from senior staff:

“There were times when I felt completely lost and overwhelmed because there wasn’t enough support from senior staff. It made an already tough job even harder.” (MIMG 11)

When support systems are weak or absent, interns may struggle to find solutions to complex medical issues, leading to increased anxiety and a sense of inadequacy. The pressure to perform well despite these challenges can contribute significantly to burnout.

The emotional demands of patient care are another critical factor contributing to stress and burnout among interns. They must navigate the delicate balance between maintaining professional detachment and providing compassionate care. This balancing act becomes particularly challenging when faced with patients’ suffering and the accompanying emotional responses from their families. MIMG 4 shared her experience of emotional exhaustion due to these demands:

“The emotional demands of patient care were overwhelming. It was hard to keep going when you constantly see people in pain and distress.” (MIMG 4)

Constant exposure to suffering can lead to emotional exhaustion, characterised by feelings of helplessness and emotional detachment from work. This state of emotional exhaustion is a core component of burnout and significantly impacts interns’ ability to engage empathetically with patients.

In conclusion, stress and burnout are pervasive challenges that MIMGs face during their internships. Long working hours, heavy patient loads, the emotional demands of patient care, and the lack of adequate support from senior staff all contribute to the high level of stress and burnout experienced by these interns.

#### Subtheme 2: Discrimination.

Feeling discriminated against, isolated, and lonely is a common experience among MIMGs during their internships, especially for those who face discrimination, are unfamiliar with the local case presentation style, or struggle with clinical note-writing. These feelings of isolation can can be exacerbated by being away from family and friends and by the demanding nature of the internship. The absence of a social support network and integration challenges further heighten these feelings, making it challenging for interns to deal with their multifaceted challenges.

Many MIMGs have reported experiencing subtle and overt forms of discrimination due to their international medical education. This discrimination often manifests itself as a lack of trust or confidence from colleagues and supervisors who may question the quality of their training. This skepticism can leave MIMGs feeling marginalized and isolated in their professional environment. MIMG 5 shared their experience with discrimination:

“I often felt that my colleagues and even some supervisors looked down on my education because I studied Indonesia. It was as if they doubted my abilities initially, which made me feel very isolated.” (MIMG 5)

MIMG 7 discussed the compounded sense of isolation they felt, not only because they were unfamiliar with local practices but also because of the subtle discrimination they experienced:

“I felt like I was always a step behind because I wasn’t familiar with the local practices. On top of that, there was this unspoken bias against my international training. It made connecting with others and feeling like part of the team hard.” (MIMG 7)

MIMG 10 and MIMG 11 emphasised the lack of support and understanding from peers, which led to profound feelings of loneliness:

“Most of my peers didn’t understand the challenges I was facing. They had their own cliques and support systems, and I often felt left out and lonely because I was struggling to keep up with the local standards.” (MIMG 10)

“Being away from my support network back home was tough. On top of that, dealing with the subtle discrimination because I graduated abroad made it even harder. It felt like a constant battle to prove myself, and it was incredibly isolating.” (MIMG 11)

MIMG 2 talked about the psychological impact of isolation and the feeling of not belonging:

“There were times when I questioned my decision to come back. The isolation and the feeling that I didn’t belong here because of my different training background took a huge psychological toll on me.” (MIMG 2)

This sense of being undervalued and scrutinised adds to the emotional burden, making it more difficult for MIMGs to build professional relationships and find their place within the medical team.

Additionally, MIMGs often face challenges in adapting to the local case presentation style, which can differ significantly from what they were accustomed to abroad. The nuances in case presentation, the expectations of supervisors, and the communication style within the medical team can create additional stress and a sense of inadequacy. MIMG 3 described their struggle with adapting to the local case presentation style:

“The way cases are presented here is quite different from what I was used to. I often felt out of place during rounds because I wasn’t sure if I was meeting the expectations. This made me feel very self-conscious and isolated.” (MIMG 3)

The pressure to conform to new standards while managing the inherent stress of the internship can lead to feelings of loneliness and isolation. MIMGs may hesitate to seek help or admit their difficulties, further compounding these feelings.

Writing clinical notes in a format that is both clear and acceptable to local standards is another area where MIMGs may struggle, particularly in hospitals without information systems. Differences in documentation practices can lead to confusion and an increased workload as they try to adapt to the expected format. This unfamiliarity can result in criticism from supervisors and colleagues, further isolating the intern. MIMG 9 highlighted the challenge of adapting to local clinical note-writing practices:

“I found it difficult to adapt to the style of writing clinical notes here. My notes were often critiqued, and I felt like I was constantly falling short. It was disheartening and made me feel very isolated.” (MIMG 9)

The need to continually adjust and improve their documentation practices, often without adequate guidance or support, can contribute to a sense of professional isolation.

### Theme 2: Training system challenges

The second theme focuses on the systemic challenges related to interns’ training programmes. These challenges are encapsulated in four major subthemes: policy and bureaucratic hurdles, inadequate infrastructure, low incentives, and inconsistent adherence to flexi-hour rules compared to training abroad.

#### Subtheme 3: Policy and bureaucratic hurdles.

Policy and bureaucratic hurdles present significant structural challenges for MIMGs. Obtaining the necessary documentation, registration, and other administrative requirements can be a lengthy and cumbersome process. These procedures often involve navigating complex and sometimes inefficient administrative systems, which can delay the start of clinical rotations and add to the stress of MIMGs. MIMG 3 shared their frustration with the bureaucratic process, MIMG 5 emphasised the inefficiency of the system, and MIMG 8 pointed out the lack of clear guidelines:

“The paperwork was overwhelming. It felt like I was constantly filling out forms and waiting for approvals, which delayed my ability to start rotations on time.” (MIMG 3)

“I spent weeks trying to get all the necessary documents in order. The process was slow and disorganised, which made it difficult to focus on my training.” (MIMG 5)

“There was a lot of confusion about what documents were needed and where to submit them. The lack of clear guidelines made the process very stressful.” (MIMG 8)

#### Subtheme 4: Inadequate infrastructure.

Inadequate infrastructure in hospitals and clinics is another major challenge faced by MIMGs. This includes outdated or malfunctioning equipment, insufficient medical supplies, and overcrowded facilities. These conditions can hinder their ability to provide effective patient care and negatively impact their learning experience. MIMG 7 discussed the frustration of working with inadequate equipment, MIMG 10 highlighted the shortage of medical supplies, and MIMG 6 spoke about the overcrowded facilities:

“Sometimes we had to work with equipment that was either broken or outdated. It was frustrating because it affected the quality of care we could provide.” (MIMG 7)

“There were times when we ran out of essential medical supplies, which made it difficult to perform even basic procedures. It was very demoralising.” (MIMG 10)

“The wards were often overcrowded, and it was challenging to manage so many patients with limited resources. It added to the stress and made it hard to learn effectively.” (MIMG 6)

#### Subtheme 5: Low incentives.

Low financial incentives are a critical issue for MIMGs, especially when they are burdened with student loans from studying abroad, unlike local graduates. The stipends or salaries provided during internships are often insufficient to cover living expenses and repay these loans, causing significant financial stress. MIMG 2 discussed the financial strain, MIMG 9 pointed out the disparity in earnings, and MIMG 11 highlighted the impact on her plans.

“The stipend we receive is barely enough to cover my living expenses, let alone pay back my study loans. The financial stress is overwhelming.” (MIMG 2)

“Compared to what I might earn abroad, the incentives here are quite low. It’s hard to stay motivated when you’re struggling financially.” (MIMG 9)

“The low pay makes it difficult to save for the future or even plan for additional training. It feels like a constant struggle to make ends meet.” (MIMG 11)

#### Subtheme 6: Non-compliance with Flexi-Hour Rules Compared to Studying Abroad.

Many MIMGs report that the flexi-hour rules, intended to provide a balance between work and rest, are often not adhered to in Malaysia. This non-compliance results in excessively long working hours, contributing to fatigue, burnout, and a reduced quality of life. MIMG 4 shared their experience with the non-compliance of flexi-hour rules, MIMG 7 compared the situation to their experience abroad, MIMG 8 described the impact on their health, MIMG 10 emphasised the inconsistency, and MIMG 2 highlighted that these extended hours lead to chronic fatigue, diminishing the interns’ ability to perform optimally:

“The flexi-hour rules are supposed to ensure we get adequate rest, but they are rarely followed. We end up working long hours without proper breaks.” (MIMG 4)

“When I was studying abroad, there were strict regulations about work hours, and they were enforced. Here, the rules exist but are not implemented, which makes it very tough.” (MIMG 7)

“The long hours and lack of rest take a toll on our health. I’ve seen colleagues burn out because the flexi-hour rules are not respected.” (MIMG 8)

“There’s a big gap between the policies on paper and what happens on the ground. It’s frustrating to know that the rules are there but not enforced.” (MIMG 10)

“There were days when I felt completely drained. The workload was immense, and there was little time to rest or recover.” (MIMG 2)

The lack of sufficient rest periods means that interns do not have adequate time to recuperate between shifts, leading to a cumulative effect of fatigue and decreased mental acuity. This continuous cycle of exhaustion compromises the interns’ health and increases the risk of medical errors, ultimately affecting patient safety.

### Theme 3: Working environment challenges

The third theme is work-environment challenges, encapsulated by two subthemes: communication barriers and different work cultures.

#### Subtheme 7: Communication Barriers.

Communication barriers present a significant challenge for MIMGs during their internships. These barriers stem from differences in medical terminology, difficulties in understanding abbreviations, and unclear instructions from specialists, often exacerbated by high-stress communication styles. Such issues can hinder effective communication, lead to misunderstandings, and contribute to the stress and anxiety experienced by MIMGs.

One of the primary language-related challenges for MIMGs is adjusting to the different medical terminologies used in Malaysia compared to those they encountered during their studies abroad. Medical jargon can vary significantly between countries, and terms familiar to local graduates may be entirely new to MIMGs. This discrepancy can lead to confusion and errors in understanding patient conditions, treatment plans, and medical procedures. MIMG 5 described their struggle with adapting to the local medical terminology:

“I struggled with medical terminology in Malay. While I could converse in basic Malay, explaining complex medical conditions was challenging.” (MIMG 5)

This challenge is compounded when MIMGs must quickly learn and apply these terminologies in high-pressure clinical environments. The learning curve can be steep, and the constant need to translate and interpret unfamiliar terms can slow their workflow and undermine their confidence.

In addition to different terminologies, MIMGs often struggle with understanding the abbreviations commonly used in Malaysian medical settings. Medical abbreviations are widely employed for efficiency, but they can vary greatly between countries and even between institutions within the same country. For MIMGs, unfamiliar abbreviations can significantly hinder effective communication and understanding. MIMG 3 highlighted the confusion caused by local abbreviations:

“The abbreviations used here were different from what I learnt abroad. I often had to ask for clarification, which made me feel inexperienced and slowed me down.” (MIMG 3)

The need to constantly seek clarification can be frustrating and time-consuming, adding to the overall stress of the internship. It also places MIMGs in a difficult position, where they may hesitate to ask questions for fear of appearing incompetent.

Another major issue related to language barriers is the difficulty in understanding instructions from specialists. In high-stress environments, specialists may give rapid or unclear instructions, sometimes speaking in a high tone, which can be intimidating for interns. This communication style can make it challenging for MIMGs to grasp the necessary information, leading to potential misunderstandings and errors. MIMG 6 shared their experience with unclear instructions from specialists:

“Specialists would often give instructions very quickly and sometimes in a high tone. It was hard to catch everything they said, and I was afraid of making mistakes because I didn’t fully understand their directions.” (MIMG 6)

The combination of unclear instructions and an intimidating communication style can create a stressful learning environment where MIMGs feel anxious about making errors and uncertain about their performance.

#### Subtheme 8: Different work culture.

The hierarchical nature of the medical field in Malaysia can create a significant gap between mentors and mentees. This strict hierarchy is commonly observed, and the relationships between senior doctors and interns are often formal and rigid. This structure can inhibit open communication and limit opportunities for MIMGs to seek guidance and mentorship. MIMG 1 described the challenges posed by this hierarchical structure:

“The strict hierarchy made it difficult to approach senior doctors with questions or concerns. There was always a sense of distance, and I often felt intimidated.” (MIMG 1)

This gap can leave MIMGs feeling unsupported and isolated. They may hesitate to seek help or clarification, fearing negative repercussions or appearing incompetent. The absence of a close mentor-mentee relationship can impede the learning process and negatively impact the overall internship experience.

Medical protocols in Malaysia are tailored to the local demographic and disease variants, which can differ significantly from what MIMGs are accustomed to abroad. For instance, certain tropical diseases or conditions prevalent in Malaysia may not be as common in the countries where these graduates studied. As a result, MIMGs must quickly adapt to new protocols and treatment guidelines, which can be challenging without prior exposure. MIMG 5 highlighted the difficulties in adapting to these different medical protocols:

“I had to learn about diseases and treatment protocols that were completely new to me. The learning curve was steep, and it was challenging to adapt quickly while managing a busy workload.” (MIMG 5)

The need to rapidly acquire knowledge about local medical conditions and adapt to different treatment protocols can be overwhelming. This challenge is compounded by the pressure to perform effectively in a clinical environment where mistakes can have serious consequences.

The high public demand for healthcare services in Malaysia, coupled with a general lack of respect for MIMGs, adds another layer of complexity to cultural adaptation. Patients and their families often have high expectations and can be critical or dismissive towards interns, especially those perceived as less experienced or foreign-trained. MIMG 3 shared their experience with the high public demand and lack of respect:

“Patients and their families sometimes questioned my competence because I studied abroad. It was disheartening to face such skepticism and disrespect while trying to provide the best care I could.” (MIMG 3)

This lack of respect can erode MIMGs’ confidence and contribute to feelings of inadequacy and frustration. The high demand for services also means interns are constantly under pressure to meet expectations, often with limited resources and support.

## Coping strategies to survive internship

**Fig 2:**
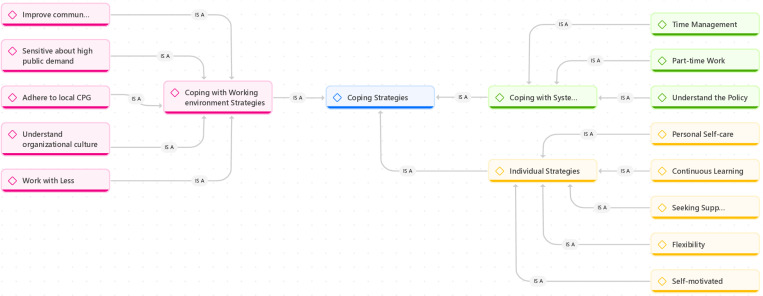
Thematic diagram illustrating coping strategies employed by Malaysian International Medical Graduates (MIMGs) to navigate their internships successfully.

The next context explored is the coping strategies that enabled them to survive and complete their training. These strategies encompass three themes: individual coping strategies, coping with the training system, and coping with the working environment (Fig 2).

### Theme 1: Individual coping strategies

Individual coping strategies, particularly resilience and the ability to adapt to new environments, are crucial for MIMGs. The capacity to recover from setbacks and adjust to new situations allows them to navigate the demanding nature of the internship. Two subthemes that elaborate on these strategies are self-motivation and flexibility

#### Subtheme 1: Self-motivated.

Maintaining a positive outlook and focusing on long-term goals can help MIMGs stay motivated. They push through difficult times by reminding themselves of their purpose and the end goal.

“I kept reminding myself why I chose this profession and focused on the end goal. It helped me stay positive and motivated.” (MIMG 6)

“Despite the challenges, I always reminded myself why I chose this path. Keeping my end goal in mind helped me stay focused and motivated, even on the toughest days. It was my passion for medicine that kept me going.” (MIMG 4)

“Staying positive was crucial for me. I would often set small, achievable goals for each day, which helped me feel a sense of accomplishment and kept my spirits high. This mindset made the difficult times more manageable and kept me resilient.” (MIMG 9)

#### Subtheme 2: Flexibility.

Being open to new experiences and willing to learn from mistakes is essential. Adapting to different clinical settings, medical protocols, and cultural practices requires flexibility.

“I realized that flexibility was key. I had to adapt quickly to new protocols and ways of working and being open-minded helped me manage these changes better.” (MIMG 3)

“Adjusting to the local work environment was challenging, especially when it came to learning the specific practices and protocols here. Initially, it felt like I had to unlearn some habits from abroad and adapt to new ways of handling cases and documentation. Embracing flexibility and actively seeking guidance from colleagues helped me integrate better and become more competent in this new setting.” (MIMG 4)

“The transition to the new work-learn environment was not easy. I had to adapt quickly to different clinical practices and communication styles that were unfamiliar to me. It required a lot of flexibility and openness to feedback. By staying proactive and continuously asking for advice, I managed to align my approach with local standards and improve my performance over time.” (MIMG 6)

#### Subtheme 3: Seeking Support.

Support from peers, mentors, and colleagues is invaluable for MIMGs. Building a network of supportive individuals can provide both emotional and practical assistance. MIMG 8 mentioned that connecting with family and friends, even remotely, offers emotional support and a sense of belonging. MIMG 10 suggested forming peer support groups with fellow interns and colleagues, which allows MIMGs to share experiences, offer advice, and provide emotional support. MIMG 4 emphasised the importance of seeking guidance from mentors who can help MIMGs navigate the complexities of the internship by offering valuable insights, encouragement, and practical advice.

“Keeping in touch with my family and friends back home was crucial. Their support kept me grounded and gave me the strength to keep going.” (MIMG 8)

“Having a group of friends who were going through the same thing made a huge difference. We supported each other and shared tips on how to handle tough situations.” (MIMG 10)

“My mentor was incredibly supportive. He provided valuable insights and advice that helped me navigate the internship successfully.” (MIMG 4)

#### Subtheme 4: Continuous Professional Development.

Engaging in continuous professional development through courses, workshops, and self-study ensures that MIMGs remain competent and confident in their medical practice. MIMG 11 participated in pre-internship crash course, which helped her to refresh her knowledge in clinical practices. Additionally, MIMG 9’s practice of reflecting on his daily experiences and learning from mistakes enables MIMGs to grow professionally and improve their practice:

“I attended crash-course workshops and read extensively to stay updated with the latest medical practices. It boosted my confidence and competence.” (MIMG 11)

“I kept a journal in Facebook where I reflected on my daily experiences. It helped me learn from my mistakes and grow as a doctor.” (MIMG 9)

#### Subtheme 5: Personal Self-Care Practices.

Maintaining a healthy work-life balance and engaging in self-care activities are essential for managing stress and avoiding burnout. MIMG 1 practised regular physical exercise and pursued hobbies to provide a necessary break from work demands, helping to reduce stress. MIMG 2 relied on prayer and other relaxation techniques to manage stress and maintain mental well-being:

“I made it a point to exercise regularly and take short breaks to relax. It really helped me manage stress.” (MIMG 1)

“Practicing mindfulness and prayer helped me stay calm and focused. It was a great way to manage the stress of the internship.” (MIMG 2)

### Theme 2: Coping with the training system

#### Subtheme 6: Effort to understand the policy and guidelines.

To cope with policy and bureaucratic hurdles, MIMGs often sought assistance from administrative staff, peers, and mentors who could guide them through the processes. Keeping detailed records and staying organized helped manage the administrative workload.

“I sought help from the administrative staff and my peers who had gone through the process before...we are contract officers. Keeping detailed records and staying organized helped me manage the paperwork more efficiently.” (MIMG 5)

“Despite the overwhelming bureaucracy, I stayed organized and kept track of all the required documents. This systematic approach helped me navigate the process more smoothly.” (MIMG 7)

“I learned to be patient and persistent. Each step forward was a small victory that kept me motivated to push through the bureaucratic red tape.” (MIMG 9)

These strategies highlight the importance of organizational skills and persistence in overcoming bureaucratic obstacles.

#### Subtheme 7: Work with less.

When dealing with inadequate infrastructure, MIMGs learned to be resourceful and innovative. They collaborated with colleagues to find alternative solutions and make the best use of available resources.

“We often had to be resourceful and find creative solutions to make do with what we had. Working closely with colleagues helped us find ways to overcome these limitations.” (MIMG 7)

“Sometimes, improvisation was key. We found ways to work around the lack of equipment and still provide the best care possible.” (MIMG 10)

“Pooling resources and collaborating with other departments helped us mitigate the impact of infrastructure deficiencies. It fostered a sense of teamwork and problem-solving.” (MIMG 3)

These responses illustrate the importance of collaboration and creativity in managing infrastructure challenges.

#### Subtheme 8: Part-time work.

To cope with financial strain, MIMGs often took on additional part-time work, applied for financial aid, and practiced strict budgeting.

“I took on part-time work as grab driver whenever off duty to manage the financial strain. Budgeting carefully also helped me make ends meet.” (MIMG 2)

“Living frugally and seeking financial assistance from bank were necessary steps to cope with the low stipend, especially to buy car.” (MIMG 4)

“Joining financial literacy programs helped me manage my finances better and plan for the future despite the low pay.” (MIMG 6)

These strategies demonstrate the proactive steps MIMGs take to manage their finances and reduce financial stress.

#### Subtheme 9: Time management.

MIMGs focused on time management and prioritised rest whenever possible to cope with the long working hours and lack of rest. They also advocated for their rights and sought support from peers and supervisors to ensure fair treatment.

“I focused on managing my time effectively and prioritized rest whenever possible. I also spoke up about the need for fair treatment and sought support from my peers and supervisors.” (MIMG 4)

“Effective time management and advocating for better working conditions were crucial. We needed to ensure our health was not compromised.” (MIMG 7)

“Forming a support network with colleagues helped us collectively address issues related to work hours and seek solutions together.” (MIMG 8)

These approaches emphasize the importance of self-advocacy and collective action in addressing work-hour challenges.

### Theme 3: Coping with working environment culture

Adapting and coping with the working environment’s culture is a multifaceted challenge that requires MIMGs to adjust to new hierarchical structures, medical protocols, and local public expectations.

#### Subtheme 10: Understand Organization Culture.

To bridge the gap created by the hierarchical structure, MIMGs sought to establish respectful and open communication channels with their mentors. They also looked for unofficial mentors among more approachable senior colleagues.

“I tried to establish a respectful relationship with my mentor and sought guidance from more approachable senior colleagues who could offer support.” (MIMG 1)

“Finding unofficial mentors, if you know Dr Iliana Medicorp who were willing to guide me informally helped bridge the hierarchical gap and provided valuable insights.” (MIMG 3)

“Building trust through consistent performance and respectful communication helped me gain the support of my mentor.” (MIMG 5)

These responses highlight the importance of building trust and seeking informal mentorship to navigate hierarchical challenges.

#### Subtheme 11: Adhere to local clinical practice guidelines.

MIMGs engaged in self-directed learning and participated in local training sessions to adapt to different medical protocols. They also sought advice from local colleagues who were familiar with the prevalent diseases and treatment protocols.

“I spent extra time studying local disease patterns and protocols. Attending local training sessions also helped me adapt more quickly.” (MIMG 2)

“Seeking advice from local colleagues who were experienced with the prevalent diseases and protocols was invaluable. Their insights helped me adjust my approach.” (MIMG 5)

“Self-directed learning and collaboration with experienced local doctors helped me understand and adapt to the different medical protocols.” (MIMG 6)

These strategies emphasize the importance of continuous learning and collaboration in adapting to new medical protocols.

#### Subtheme 12: Sensitive about high public demand.

MIMGs focused on building their professional reputation through consistent performance and patient-centred care to cope with high public demand and the lack of respect. They also educated patients about their qualifications and training to build trust and respect.

“I focused on providing the best care possible and building my professional reputation. Educating patients about my qualifications helped build trust.” (MIMG 3)

“Demonstrating competence and empathy in patient care helped me gain the respect of patients and their families over time.” (MIMG 9)

“Consistency in my work and taking the time to communicate effectively with patients gradually earned their respect and trust.” (MIMG 10)

These responses highlight the importance of professionalism and patient education in gaining respect and managing public demand.

#### Subtheme 13: Improve communication skill.

Language barriers, including unfamiliar medical terminology, difficulties with abbreviations, and unclear instructions from specialists, pose significant challenges for MIMGs during their internships in Malaysia, particularly for those who graduated from Indonesia. These barriers can lead to misunderstandings, errors, and increased stress, affecting both patient care and professional relationships. Here are some strategies MIMGs employ to overcome these challenges:

“The medical terminology here was quite different from what I learned abroad. To cope, I created a glossary of terms and abbreviations notes. I would review it regularly and ask my colleagues for explanations whenever I encountered unfamiliar terms. This proactive approach helped me gradually become more comfortable with the local terminology.” (MIMG 1)

“Understanding the local medical jargon and slang was one of the toughest challenges for me. Sabahan working at Kelantan, can you imagine? Kelantanese dialect is tough to understand..I will ask senior interns who could explain the terms in simpler language. I also made a habit of noting down any new terms I came across and looked them up later to build my understanding.” (MIMG 7)

Providing a targeted language training short course focused on the medical terminology and abbreviations used in Malaysia could help MIMGs become more familiar and comfortable with local jargon.

In addition to different terminologies, MIMGs often face difficulties in understanding the abbreviations commonly used in Malaysian medical settings. Medical abbreviations are widely used for efficiency, but they can vary significantly between countries and institutions within the same country. For MIMGs, unfamiliar abbreviations can hinder substantially effective communication and understanding.

“The abbreviations used here were different from what I learnt abroad. I often had to ask for clarification, which made me feel inexperienced and slowed me down.” (MIMG 3)

“The abbreviations and specific terms used in clinical notes were overwhelming at first. I reached out to my peers and supervisors to clarify any confusion and made use of online medical dictionaries. I also participated in team discussions and asked questions during meetings to get familiar with the common terminology used in practice.” (MIMG 9)

Another major issue related to language barriers is the difficulty of understanding instructions from specialists. In high-stress environments, specialists may give rapid or unclear instructions, sometimes speaking in a high tone, which can be intimidating for interns. This communication style can make it challenging for MIMGs to grasp the necessary information, leading to potential misunderstandings and errors.

“Having a supervisor who took the time to explain things clearly made a huge difference. They didn’t get frustrated when I asked questions, which helped me learn faster and feel less anxious.” (MIMG 8)

Encouraging specialists and supervisors to communicate clearly and patiently, avoiding the use of high tones, can create a more supportive learning environment. Clear and concise instructions can help reduce misunderstandings and build confidence among MIMGs.

## Discussion

The findings highlight the multifaceted nature of the challenges faced by MIMGs and underscore the need for systemic improvements. The study reveals that stress and burnout, bureaucratic inefficiencies, and cultural adaptation issues significantly impact the internship experience. Addressing these challenges through streamlined administrative processes, enhanced infrastructure, financial support, and cultural competence training can improve the overall training environment for MIMGs. The study also emphasises the importance of resilience and peer support in managing individual stressors.

### Individual challenges

One of the most pronounced individual challenges identified in this study is stress and burnout. MIMGs reported high levels of stress driven by demanding work hours, heavy patient loads, and the emotional toll associated with patient care. These findings corroborate existing research that emphasises the widespread nature of stress and burnout among IMGs worldwide [37–40]. Interns often find themselves in high-pressure environments with insufficient time for rest and personal recuperation, exacerbating feelings of exhaustion and emotional strain [41].

MIMGs employed various coping strategies to manage these challenges. Resilience emerged as a crucial factor, with many interns relying on their inner strength and adaptability to navigate stressful situations. Peer support was also vital, as interns formed support networks to share experiences, offer encouragement, and provide practical advice. Additionally, self-care practices, such as regular exercise and mindfulness techniques, played a significant role in mitigating the effects of stress and promoting mental well-being. These findings suggest that enhancing support systems and integrating self-care practices into training programs could be beneficial in reducing burnout and improving the overall internship experience.

### Training system challenges

MIMGs faced significant hurdles related to the training system, including bureaucratic inefficiencies, inadequate infrastructure, and low financial incentives. The study revealed that complex administrative procedures and delays in registration created obstacles that hindered the smooth initiation and progression of internships. Inadequate infrastructure, such as outdated equipment and insufficient resources, further compounded these challenges, affecting the quality of medical training and patient care. Low financial incentives also contributed to the stress experienced by MIMGs, as many struggled with financial burdens, including the repayment of student loans.

These challenges align with existing literature that highlights bureaucratic inefficiencies and infrastructural issues as common barriers in medical training environments [42,43]. To address these issues, MIMGs adopted strategies such as improved organisational skills, persistence in navigating bureaucratic processes, and seeking support from administrative staff and peers. However, systemic changes are necessary to alleviate these challenges on a broader scale. Streamlining administrative procedures, investing in modern infrastructure, and increasing financial support for interns could significantly enhance the training experience and reduce stress related to systemic issues.

### Working environment challenges

Cultural adaptation and language barriers emerged as significant challenges within the working environment. MIMGs experienced difficulties in adjusting to the hierarchical structures prevalent in Malaysian medical training. These hierarchical dynamics often created communication gaps between mentors and mentees, affecting the quality of mentorship and support. This finding is consistent with studies that indicate hierarchical structures in medical settings can impede effective mentoring and professional development [44,45]. MIMGs coped by seeking informal mentorship and focussing on building professional relationships through consistent performance and respectful communication.

Language barriers also posed a significant challenge, particularly with unfamiliar medical terminology, abbreviations, and unclear instructions from specialists. The study supports existing research that demonstrates the impact of language barriers on medical practice and learning outcomes [46,47]. MIMGs addressed these barriers by seeking clarification from colleagues and supervisors and utilising peer support networks to navigate dialect challenges. Addressing these language and cultural barriers through targeted training and clear communication can improve the integration and effectiveness of MIMGs within the local medical environment.

## Limitations of the study

This study has several limitations that should be acknowledged. First, the sample size of eleven participants, while providing valuable insights, may not fully represent the diverse experiences of all MIMGs. The relatively small sample size limits the generalisability of the findings to the broader population of MIMGs. Second, the study relies on self-reported data, which is subject to recall bias and social desirability bias. Participants may have presented their experiences in a more favourable light or may have omitted certain challenges due to the nature of self-reporting. Lastly, the findings are specific to the Malaysian context and may not be directly applicable to other countries or medical systems. Variations in healthcare systems, cultural norms, and training structures may influence the experiences of MIMGs in different settings.

### Recommendations for future research

Future research should address these limitations by employing larger and more diverse sample sizes to enhance the generalisability of findings. Additionally, employing mixed-methods approaches that combine quantitative and qualitative data could provide a more comprehensive understanding of the challenges faced by MIMGs. Longitudinal studies that track the experiences of MIMGs over time could offer insights into the long-term effects of these challenges and the effectiveness of coping strategies. Furthermore, comparative studies across different countries and medical systems could identify universal and context-specific challenges, leading to more targeted interventions. Lastly, future research should explore the impact of systemic changes, such as streamlined administrative processes and enhanced support systems, on the experiences and outcomes of MIMGs.

## Conclusions

Unique challenges from differences in educational backgrounds, cultural and language barriers, and varying levels of support within the healthcare environment mark the journey of MIMGs through the medical internship system in Malaysia. These factors, compounded by the complexities of integrating into a healthcare system that differs significantly from the one they trained in abroad, often result in a strenuous internship experience for MIMGs. Addressing these challenges requires a multi-faceted approach, including tailored support systems, enhanced mentorship, and policy reforms aimed at creating a more inclusive and conducive training environment. By understanding and addressing the specific needs of MIMGs, Malaysia can not only improve the internship experience for these graduates but also strengthen the overall quality of its healthcare workforce, ultimately contributing to better patient outcomes and a more resilient healthcare system.

## Abbreviations

MIMGs: Malaysian International Medical Graduates
MMC: Malaysian Medical council
MOH: Malaysia Ministry of Health.
